# School Closure and Children in the Outbreak of COVID-19

**DOI:** 10.2174/1745017902016010189

**Published:** 2020-08-18

**Authors:** Donatella Rita Petretto, Ilaria Masala, Carmelo Masala

**Affiliations:** 1 Department of Pedagogy, Philosophy and Psychology, University of Cagliari, Cagliari, Italy; 2 Studio Medico Neuropsicologico Masala Pistis, Piras, 09047Selargius, Italy

**Keywords:** School closure, Distance learning, COVID-19, Outbreak lockdown, Learning disabilities, Psychological consequences

## Abstract

School closure and home confinement are two of the measures of lockdown chosen by governments and policymakers all over the world to prevent and limit the spread of the infection of COVID-19. There is still an open debate about the real effect of school closure on the reduction of risk of infection on children and the risk of infection on with other age groups (parents, grandparents and others). There is an agreement on the effect of school closure in reducing and delaying the peak of the outbreak. In this Editorial, starting from the ongoing Italian experience, we discuss direct and indirect effects of school closure on children’s psychological health and learning. We also highlight the need for an “on peace time” planning of measures and strategies necessary to face the direct and indirect effect of this outbreak and other outbreaks, on children’s psychological health.

School closure and home confinement are two of the measures of lockdown chosen by governments and policymakers all over the world to prevent and limit the spread of COVID-19. There is still an open debate about the real effect of School Closure on the reduction of risk of infection on children and on the risk of infection on other age levels relatives (parents, grandparents and others) [1-3]_._ There is an agreement on the effect of school closure in reducing and delaying the peak of the outbreak [1]. Different countries have chosen different points of school frequency reduction. Still, in over 100 countries about one-half of the children in the world are not attending school during COVID-19 outbreak, as of 18 March 2020 and in 188 countries as of 8 April 2020 [1]. There is an open debate also about the risks of infection and the direct and indirect consequences of COVID-19 in children. About direct consequences, new epidemiological studies are describing known and new effects on children and only in the future, epidemiological studies will give us a clear picture of the direct consequences of COVID-19 on children’s health. By now, in general population, we know that the lower the age, the lower the risk of infection and the severity of pathology, and the higher the number of comorbidities, the higher the risk of infection and severity of pathology. But in children there could also be some peculiar clinical signs and symptoms different from adults, children of all ages are susceptible but infants had more severe outcomes, as stated in Chinese data [4-6].

But what are the indirect consequences of COVID-19 on children? Some previous and recent papers highlighted the psychological effects of pandemic on children and the effect on different aspects of health and wellbeing, like developing negative lifestyles on eating, the reduction of movements and physical activities, sleep disturbances and other psychological consequences [7-10]. Some authors described the psycho-logical effects of quarantine and social distancing in children and in adults in more detail. They found emotional disturbance, depression, stress, low mood, irritability, insomnia, posttraumatic stress symptoms and attentional deficit [2,11]. Other papers focused on a higher risk of home abuse and maltreatment during home confinement. Previous studies on other kind of pandemic, like Ebola, described a higher frequency of juvenile pregnancies after the outbreak, due to forms of family sexual abuse or other kinds of home abuse [12]. Other articles highlighted the economic consequences of the outbreak lockdown and they put attention to the countries and situation where schools are the main source of food for students [8,10, 13, 14]. Other papers focused on the role of the school in various other domains in the child’s life which negatively influenced during school closure: social contacts and social development. They are the place where sport and physical activities are provided, and in some cases, it is also the place where healthcare and mental health services are provided [8]. Last, but not least, some other authors focused their work on a prevention approach and they described the need for a preliminary “on peace time” plan to promote wellbeing in children and to prevent negative effects and post-traumatic disorders [7, 10, 15,16]. Unfortunately, even if some papers described short- and long- term effects of outbreak and lockdown in children, and even if children’s life changed completely in the acute phase of pandemic, due to social isolation, home confinement and school closure, little attention is paid on them, on what they felt during acute phase of outbreak and lockdown. Moreover, little attention is paid on what they are feeling now during new phases of progressive reduction of lockdown, neither they are in center of attention when planning new phases of “reconstruction” after pandemic. We agree with Wang and colleagues that “children have little voices to advocate their own needs” [10].

In this paper, we’d like to propose some issues of analysis starting from the ongoing experience in Italy, that is one of the first European countries that had to fight against COVID-19 outbreak.

In Italy, since the first ten days of March, children, preadolescents and adolescents stayed home from school. They will come back to school only in September 2020 and by now, it is not clear if all children will come back to school together. The Italian Minister of Education is planning the re-opening the school taking into account all measures needed to prevent infection.

During acute phase of the pandemic, children in Italy stayed home for about two months, with a huge reduction of direct contact with friends, grandparents and other relatives. Children’s life changed completely in the acute phase of pandemic, due to social isolation, home confinement and school closure. Different from experience in other outbreaks, in Italy, the school closure was generally not related to a mixing with others and social contacts, because children stayed home all the time [11]. The use of new technologies (like personal computers, tablets, and smartphones) made possible indirect contacts, but due to “digital divide” and economic issues, not every child has these new devices. In each country, as the previous and new socioeconomic levels of families range from high to very low, the quality of housing solutions, the size of houses and spaces available for each person in the house change a lot from one family to another. Even if in Italy and other countries, governments are giving economic help to families, economic consequences of the outbreak are one of the worst indirect effects, now and for the near future [13]. So, social experiences and social contacts of children changed completely during this acute phase of pandemic.

Also, the children’s learning changed completely in the acute phase of pandemic, due to “distance learning”. In the first phase of the outbreak, in Italy, there was a complete school closure and then the school Minister, school directors and teachers organized different kinds of “distance learning measures”, based on “home based-distance learning”. Now they are organizing different kinds of distance assessment for formative and final formal evaluation of learning. So, schools go completely on “learning electronic platforms”. This is a positive aspect and a way to cope with the outbreak and the risk of infection, and it is the consequence of high civic sense of teachers in trying to keep connected with their students. But there are positive aspects and some other issues to be considered. Economic issues and “digital divide” are to be considered because if learning depends on the availability of electronic devices (like personal computers, tablets and smartphone) not every child has any one of them. Moreover, another critical aspect is the availability of internet connection. Again, Minister of Education and other sources (like schools and municipalities, or private sources) give some economic help to families and students to buy devices and to pay internet connection. Another aspect is that the “home-based distance learning” is based on the presence of parents, mainly the mothers, that during the acute phase of outbreak were at home. The lower the age of the child, the higher the burden of “home-based distance learning” on family members, mainly because the younger child needs more support in keeping in touch with the school *via* computer and technological devices and they also need more support during learning tasks. In the acute phase of pandemic, only workers in the “essential services” go to work, the others stayed home and worked on “smart working mode”. Unfortunately, some workers stayed at home because they lost their jobs due to economic crisis. Nevertheless, mainly the mothers support children during “home-based distance learning” and during other kinds of “home learning tasks”. But in the other phases of outbreak, the return to work of parents puts a higher burden on other family members (like grandparents, when present) or directly on children. Moreover, it is important to consider and assess if “distance learning” is really able to convey all the aspects of learning and education in school and what kind of measures and strategies are needed to increase the quality of “distance learning” if Government and the Minister of Education will decide to continue to use “distance learning” for a longer time. In our opinion, it is very important to discuss, promote and increase the quality of “distance learning” and its inclusiveness and to support teachers and other school professionals (like educators and psychologists) in running the process. But it is also important to support families that now have the main burden of learning.

Elsewhere, we have talked about special needs for children with learning disabilities and other special educational needs during school closure, home confinement and “Home-based distance learning”. It is very important to discuss the impact of distance learning on different neuropsychological profiles and different neurodevelopmental disorders, capitalizing on previous studies on this topic [17]. And what about learning of children with attentional disorders and the impact of “distance learning”? [18] and what about learning of children with visuospatial disorders? With working memory impairments? Again, especially for children with learning disabilities and other special educational needs, the complete burden of “home-based distance learning”, of reasonable accommodation and personalization in learning, is on family members. And again, in the other phases of the outbreak, the return to work of parents puts a higher burden on other family members (grandparents, older siblings, babysitters, when present) or directly on children.

And what about other consequences on children’s health and mental health? And what about consequences on general health of children? Home confinement could have a negative effect on diet, physical activities and healthy lifestyles.

According to Poletti and Raballo [3], we believe that it is mandatory to have a clear picture of psychological consequences of the outbreak on all children, both for children that had previous psychological disorders and children that developed new ones during this time. And it is very important to support them to face all the phases of pandemic and to develop adaptive coping strategies.

Starting from the ongoing experience of pandemic of COVID-19, we highlight the importance of international and national attention on children’s health, during all the phase of outbreak of COVID-19 and, in the near future, in planning “on peace time” programs to run future outbreaks and their consequences on children [15]. We believe that, even in complex situations, awareness of different aspects of a situation is the first step, then it is necessary to take all the aspects into account [19, 20].

Government, scientific societies, stakeholders and health policy makers have a central role in planning and implementing specific intervention to protect children and to promote their wellbeing, even in emergency situations. Health policy makers, school directors, teachers, educators, and administrators should collaborate with communities and families to minimize the effects of school closure and to support children to overcome the outbreak, to cope with the different phases of reduction of lockdown, to control medium and long term psychological consequences, to promote their wellbeing and their learning and development.
